# A transformative interprofessional model for geriatric oral health care - A proposed working model

**DOI:** 10.3389/fdmed.2026.1757007

**Published:** 2026-03-05

**Authors:** Ashwini Tumkur Shivakumar, Sowmya Srinivas, Sowmya Halasabalu Kalgeri, M. Kishor, Shilpa Avarebeel

**Affiliations:** 1 Department of Conservative Dentistry and Endodontics, JSS Dental College and Hospital, JSS Academy of Higher Education and Research, Mysore, Karnataka, India; 2 Department of Prosthodontics and Crown and Bridge, JSS Dental College and Hospital, JSS Academy of Higher Education and Research, Mysore, Karnataka, India; 3 Department of Psychiatry, JSS Medical College and Hospital, JSS Academy of Higher Education and Research, Mysore, Karnataka, India; 4 Department of Geriatric Medicine, JSS Medical College and Hospital, JSS Academy of Higher Education and Research, Mysore, Karnataka, India

**Keywords:** comprehensive geriatric integrated care model, geriatric dental clinic, interprofessional collaboration, interprofessional dental care, IP model, multidisciplinary care

## Abstract

**Background/rationale:**

The rapid global expansion of the geriatric population has intensified the burden of chronic diseases, functional decline, and healthcare utilization. Oral health, despite its well-established links with systemic health, nutrition, cognition, and quality of life, remains marginalized within conventional geriatric care models. Fragmented health systems and discipline-specific silos have perpetuated inequities in oral healthcare access for older adults.

**Objective:**

This Perspective article proposes a transformative interprofessional working model designed to integrate oral healthcare into comprehensive geriatric services, thereby addressing oral health disparities and promoting holistic, person-centred care for the ageing population.

**Model description:**

The proposed model brings together dentists, geriatricians, psychiatrists, nutritionists, clinical pharmacists, and occupational therapists within a cohesive care unit. It emphasizes structured interprofessional collaboration, clearly defined roles, and shared care pathways. In addition to clinical integration, the model prioritizes education and capacity-building for caregivers, healthcare workers, and students. Key components include geriatric-friendly clinic design, community and caregiver education modules, home-based oral healthcare, tele-dentistry, and the use of portable dental units to improve access for functionally dependent older adults.

**Expected outcomes:**

The model demonstrates feasibility in real-world settings, with anticipated outcomes including improved patient satisfaction, earlier identification of oral–systemic risks, reduced unnecessary medical referrals, enhanced continuity of care, and better oral and general health-related quality of life among older adults.

**Implications:**

By reframing oral health as an integral component of healthy ageing, this interprofessional model offers a scalable and adaptable framework for academic institutions, policymakers, and healthcare systems. Its adoption could significantly reduce oral health disparities and contribute to sustainable, preventive, and integrated geriatric care globally.

**Rationale for the proposed interprofessional model:**

Older adults commonly present with multimorbidity, polypharmacy, and functional limitations that directly influence oral health, yet dental care remains largely disconnected from geriatric services. This fragmentation leads to delayed diagnosis, reactive treatment, and preventable declines in quality of life. An interprofessional model integrating dental and geriatric care enables early risk identification, coordinated prevention, and continuity of care. By addressing shared biological, functional, and social determinants, this approach repositions oral health as an essential component of holistic, person-centred healthy ageing.

## Introduction

The world is undergoing an unprecedented demographic transition characterized by increased life expectancy and a rapidly expanding older population. According to United Nations projections, by 2050, one in six individuals globally will be aged 65 years or older, with the fastest growth occurring in low- and middle-income countries, thereby placing substantial strain on existing healthcare systems (1). While healthy ageing has emerged as a global public health priority, oral health—an essential determinant of functional ability, nutrition, communication, and psychosocial well-being—remains consistently neglected in geriatric care models (2, 3). Older adults disproportionately experience oral health disparities, including higher prevalence of dental caries, periodontal disease, edentulism, xerostomia, and oral mucosal disorders, often compounded by multimorbidity, polypharmacy, socioeconomic disadvantage, and limited access to dental services (4, 5).

Despite robust evidence linking poor oral health with systemic conditions such as diabetes mellitus, cardiovascular disease, respiratory infections, frailty, and cognitive decline, oral healthcare continues to be siloed from mainstream medical and geriatric services (6). This separation is reinforced by fragmented health systems, inadequate oral health coverage within universal health care frameworks, and limited interprofessional training, resulting in delayed diagnosis, suboptimal disease management, and diminished quality of life among older adults (4). The World Health Organization and the FDI World Dental Federation have emphasized that failure to integrate oral health into general healthcare undermines efforts toward healthy ageing and exacerbates health inequities (6, 7).

Consequently, there is an urgent need to transition from discipline-centric models toward integrated, interprofessional frameworks that embed oral health within geriatric medicine, primary care, nutrition, nursing, and allied health services (8). Such health-system integration supported by shared care pathways, collaborative education, and preventive strategies has the potential to improve early risk identification, enhance chronic disease management, and promote person-centred, holistic geriatric care aligned with the life-course approach to health (9).

## The interprofessional model

**Figure 1:**
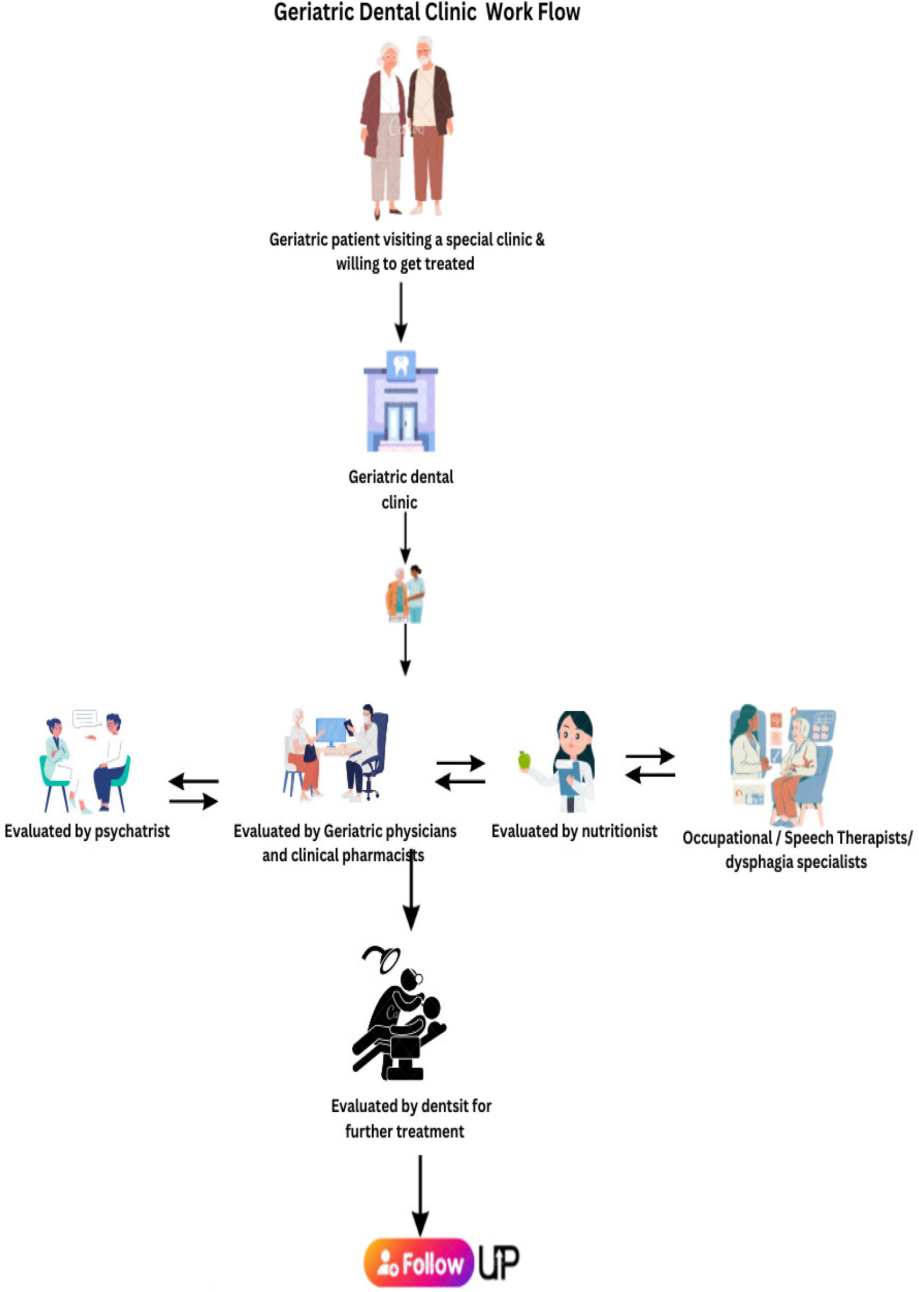
Workflow of the geriatric clinic.

The proposed Interprofessional Geriatric Oral Health Care Model seeks to redefine dental service delivery for elderly populations through collaboration among diverse healthcare professionals (Figure 1).

## Core team composition and roles and responsibilities

**Figure 2:**
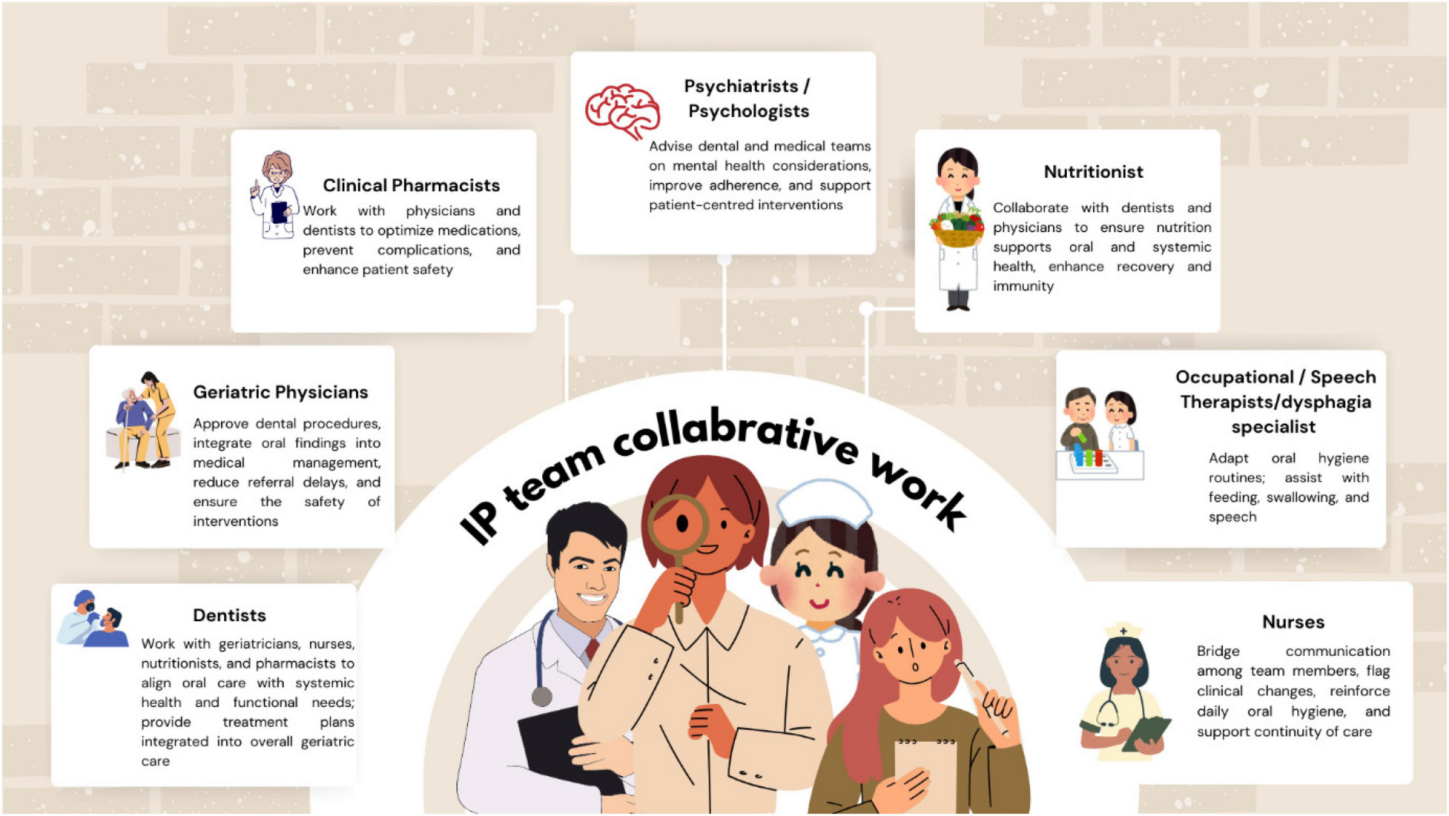
Collaborative work of an interprofessional team.

A collaborative framework ensures continuity of care that respects the physical, cognitive, and social dimensions of aging. The core team members include (Figure 2; Table 1).

**Table 1:** Interprofessional geriatric oral health care model—team roles and collaboration.

Team Member	Primary Roles/Responsibilities	Collaborative Interactions/Impact	Impact
Dentists (Conservative, Prosthodontists, Periodontists, Oral Surgeons, Community Dentists)	Comprehensive oral assessment; preventive and restorative care; prosthetic rehabilitation; manage periodontal/systemic links; outreach and education	Work with geriatricians, nurses, nutritionists, and pharmacists to align oral care with systemic health and functional needs; provide treatment plans integrated into overall geriatric care	The dental team works closely with geriatric physicians, nurses, nutritionists, and allied health professionals to align oral interventions with systemic health goals, functional rehabilitation, and patient safety.
Geriatric Physicians	Comprehensive geriatric assessment; evaluate medical fitness; manage comorbidities; coordinate care	Approve dental procedures, integrate oral findings into medical management, reduce referral delays, and ensure the safety of interventions	Geriatricians ensure that oral care decisions are medically informed, safe, and seamlessly integrated into each patient's overall care plan
Nurses	Oral screening, caregiver education, adherence monitoring, documentation	Bridge communication among team members, flag clinical changes, reinforce daily oral hygiene, and support continuity of care	Nurses bridge clinical silos, improving early detection, adherence, and patient-centred care.
Psychiatrists/Psychologists	Cognitive and mood assessment; early detection of dementia/depression; behavioural management	Advise dental and medical teams on mental health considerations, improve adherence, and support patient-centred interventions	Early detection and integrated management of cognitive or psychiatric issues support mental health, treatment adherence, and overall well-being
Nutritionists	Assess dietary intake; develop individualized nutrition plans; support mastication and digestion	Collaborate with dentists and physicians to ensure nutrition supports oral and systemic health, enhance recovery and immunity	Proper nutrition supports oral healing, systemic health, and quality of life
Clinical Pharmacists	Medication review; manage polypharmacy; monitor drug interactions and oral side effects	Work with physicians and dentists to optimize medications, prevent complications, and enhance patient safety	Pharmacists optimize therapeutic outcomes, prevent polypharmacy-related complications, and enhance patient safety
Occupational/Speech Therapists/dysphagia specialist	Adapt oral hygiene routines; assist with feeding, swallowing, and speech	Ensure functional independence, prevent malnutrition and aspiration, and improve quality of life.	These specialists enhance functional outcomes, prevent complications.adds up to quality of life

**Dentists:** In a geriatric clinic, dentists play a pivotal role in promoting oral health and well-being among older adults through comprehensive assessment, prevention, and coordinated care. Oral care also contributes to patients' systemic health.

**Conservative dentists** focus on preserving natural teeth through minimally invasive restorative treatments and root canal therapies, reducing infection risks. **Prosthodontists** rehabilitate missing dentition with dentures or implants to restore mastication, speech, and aesthetics, enhancing nutrition and quality of life. **Periodontists** manage periodontal disease and its systemic implications, emphasizing the control of inflammation, which contributes to patients' overall health. **Oral surgeons** address complex extractions, oral pathology, and pre-prosthetic surgeries, ensuring medical safety in medically compromised patients. **Community dentists** lead outreach, screening, and preventive programs for institutionalized or homebound elders.

**Collaborative impact**: The dental team works with physicians, nutritionists, speech therapists, and geriatricians to develop holistic, patient-centered care plans. Their coordinated efforts ensure timely diagnosis, functional rehabilitation, and maintenance of oral health as an integral component of healthy ageing (5).

**Geriatric physician:** They play a vital role in assessing the physical fitness of patients and monitoring their systemic health to ensure safe and effective dental care. Evaluating medical fitness for dental procedures and coordinating with dentists to ensure safe treatment planning. Integrating oral health findings into broader medical management, reducing unnecessary referrals, and healthcare delays. Overseeing continuity of care by facilitating on-site medical consultations during dental visits, minimizing patient stress and logistical barriers. Aligning oral care plans with systemic health priorities, enhancing patient safety, and improving treatment efficacy (12).

Having all essential healthcare services available within a single facility allows for seamless coordination between medical and dental teams. This integration enables dentists to make informed treatment decisions promptly, as medical fitness evaluations and consent can be obtained on-site. This helps to minimize the need for elderly patients to travel between different clinics, reducing stress and treatment delays. Moreover, it fosters timely interventions and enhances patient safety by ensuring that oral care plans are designed in alignment with the individual's overall health status. This collaborative and patient-centred approach exemplifies the essence of comprehensive geriatric care, where convenience, efficiency, and holistic well-being are prioritized (13, 14).

**Impact:** Geriatricians ensure that oral care decisions are medically informed, safe, and seamlessly integrated into each patient's overall care plan.

**Psychiatrists/Psychologists:** They play a crucial role in the early evaluation and identification of cognitive disorders such as dementia and depression among older adults. Routine dental visits provide an opportunity to observe behavioural changes, memory lapses, or altered communication patterns that may indicate early cognitive decline. Early recognition during these interactions enables timely intervention, which is vital for maintaining mental health and overall well-being. Many elderly patients may be hesitant to consult psychiatrists or psychologists due to social stigma or lack of awareness; hence, incorporating basic cognitive screening within dental settings offers a discreet and accessible avenue for detection (6).

**Impact:** Early detection and integrated management of cognitive or psychiatric issues support mental health, treatment adherence, and overall well-being.

**Nutritionists** play a key role in counselling and designing individualized dietary plans for geriatric patients, considering their systemic health conditions and specific nutritional needs. They assist in formulating balanced diets that support overall health, enhance immunity, and promote better recovery outcomes. For denture wearers, nutritionists design meal plans that are easy to chew and digest while ensuring adequate intake of essential macro and micronutrients that may otherwise be lacking due to dietary limitations. By working closely with dental and medical professionals, nutritionists help maintain optimal nutrition, prevent deficiencies, and improve the overall quality of life of elderly patients (7).

**Impact:** Proper nutrition supports oral healing, systemic health, and quality of life.

**Clinical pharmacists** play a vital role in the multidisciplinary care of geriatric patients by ensuring the safety and effective use of medications. They review and manage all prescribed drugs, paying close attention to potential drug–drug and drug–disease interactions, which are particularly common in older adults with multiple comorbidities and polypharmacy. Clinical pharmacists also monitor for adverse drug reactions and adjust dosages based on renal or hepatic function to minimize side effects. Their expertise supports rational prescribing practices and enhances medication adherence through patient education and counselling. By collaborating closely with physicians and dentists, clinical pharmacists help optimize therapeutic outcomes, prevent polypharmacy-related complications, and promote patient safety—an essential component of comprehensive geriatric care (8).

**Impact:** Pharmacists optimize therapeutic outcomes, prevent polypharmacy-related complications, and enhance patient safety.

**Nurses:** Nurses act as primary integrators and continuity providers within the team. They can conduct routine oral health screenings once trained, identify early signs of disease, and document observations in shared care records. Reinforce daily oral hygiene practices and provide caregiver training for home care. Monitor adherence to oral care and medical regimens, flagging changes in functional or cognitive status for timely intervention. Facilitate communication among team members, patients, and families to ensure cohesive care delivery (15).

**Impact:** Nurses bridge clinical silos, improving early detection, adherence, and patient-centred care.

**Allied Health Professionals** (Occupational and Speech Therapists):

Occupational therapists assist patients in adapting oral hygiene routines to physical limitations, ensuring independence in daily care. Speech therapists evaluate and improve articulation, swallowing, and feeding, particularly in patients with prosthetic devices or neurological impairments. A **dysphagia specialist** is essential for assessing and managing swallowing disorders common in older adults. They help prevent **malnutrition, dehydration, aspiration, and choking** through safe swallowing strategies and diet modifications. By collaborating with dentists, physicians, nutritionists, and nurses, they ensure **holistic, patient-centred care**. Their involvement improves **oral health, functional independence, and overall quality of life** in elderly patients (16).

**Impact:** These specialists enhance functional outcomes, prevent aspiration or malnutrition, and improve overall quality of life.

## A critical aspect of geriatric-interprofessional friendly clinics

**Clinic Design** Designing geriatric dental clinics requires a patient-centred approach that accommodates the physical, cognitive, and sensory needs of older adults while ensuring safety, accessibility, and efficiency. Universal and age-friendly design principles aim to reduce barriers, enhance comfort, and promote independence, consistent with WHO guidance on age-friendly health services (11, 12).

## Key design features for an interprofessional geriatric dental clinic

**Physical Accessibility** Barrier-free entryways, ramps, wide corridors, and automatic doors facilitate mobility for patients using wheelchairs, walkers, or canes. Adjustable dental chairs and supportive seating accommodate musculoskeletal limitations and ensure treatment comfort (13). Non-slip flooring and well-lit pathways minimize fall risk, critical for frail older adults (18).

**Cognitive-Friendly Environments** Clear signage, contrasting colours, and intuitive wayfinding support patients with dementia or mild cognitive impairment, reducing confusion and anxiety (19). Noise control and calm waiting areas help maintain focus and reduce agitation during treatment. Visual cues and structured layouts allow caregivers to assist patients efficiently.

**Integrated Care Spaces** Co-located medical and dental treatment rooms enable seamless collaboration among dentists, geriatricians, nurses, and allied health professionals (18). Space for caregiver participation promotes comfort, facilitates training, and supports continuity of care.

**Technology-Enabled Access** Tele-dentistry stations, portable dental units, and home-visit capabilities extend services to institutionalized or homebound elders, minimizing travel-related stress and treatment delays (20, 21).

**Safety and Infection Control** Handrails, emergency call systems, and infection control protocols ensure patient safety, particularly for medically complex individuals (18).

## Design considerations for a geriatric-friendly dental clinic

A geriatric-friendly dental clinic should be thoughtfully designed to ensure safety, comfort, accessibility, and effective communication for elderly patients. Such inclusive design helps reduce fear and physical strain, improving treatment adherence and satisfaction. The following features are essential.

**Private treatment cabins:** Individual cabins or enclosed treatment spaces should be provided to ensure privacy, dignity, and a calm environment during dental procedures, thereby reducing anxiety and enhancing patient comfort (9).
**Barrier-free access:** Clinics must incorporate wheelchair-accessible entryways, wide doorways, ramps with handrails, and anti-slip flooring to ensure safe and independent mobility for patients with physical limitations.
**Ergonomic dental chairs:** Adjustable dental chairs with stable armrests and adequate head support facilitate safe patient transfer and improve comfort for individuals with restricted mobility, joint stiffness, or musculoskeletal disorders (23).
**Sensory-friendly environment:** Dental clinics should maintain calm, well-lit spaces with minimal background noise. Clear, high-contrast signage in bold lettering should be used to support patients with visual or sensory impairment.
**Comfortable waiting areas:** Seating should be firm and positioned slightly above knee level to assist ease of standing. Waiting areas should be well-ventilated, adequately illuminated, designed with soothing colours, and kept free of clutter to reduce stress and anxiety (9, 24).
**Communication-friendly measures:** Dental professionals and staff should use transparent or clear masks to facilitate lip reading for patients with hearing impairments. Communication should be clear, slow, and delivered at a moderate pace to enhance comprehension (23).
**Emergency preparedness:** Clinics should be equipped with handrails, grab bars, and clearly accessible emergency alert systems to ensure prompt and effective response during medical emergencies (25).
**Accessible restrooms:** Washrooms should include non-slip flooring, wide door access, and appropriately positioned support bars to promote safety, independence, and convenience (23, 24).
**Specialized treatment areas:** Designated treatment spaces should include wheelchair-compatible dental setups that allow direct wheelchair positioning or easy conversion into a dental chair. This minimizes patient transfer, reduces injury risk, and improves comfort and safety for individuals with severe mobility limitations (25).

## Special considerations for cognitive and physical impairments

Patients with cognitive decline benefit from predictable spatial layouts, visual cues, and caregiver-friendly design, reducing disorientation and improving treatment adherence (18). Those with physical limitations require adjustable equipment, wider working spaces, and ergonomic supports. These design features enhance patient experience, reduce caregiver burden, and enable interprofessional collaboration, ultimately supporting holistic geriatric care (13).

## Community outreach: extending care beyond the clinic

These educational strategies create a culture of collaboration and shared responsibility in geriatric health care. To overcome access barriers, the model emphasizes community-based service delivery through.

## Home visits for immobile or dependent elderly patients

Home visits play a crucial role in extending dental care to elderly patients who are immobile, bedridden, or unable to visit the clinic due to physical, cognitive, or medical limitations. These visits ensure continuity of care and help maintain oral health as an integral component of overall well-being. During home visits, dentists and allied professionals can perform essential services such as oral examinations, oral hygiene instruction, denture adjustments or repairs, fluoride applications, and palliative management of pain or infection (10).

Portable dental equipment and mobile dental units enable clinicians to deliver safe and efficient care in the patient's home environment. This approach not only enhances access to care but also minimizes anxiety and transportation-related challenges for both patients and caregivers. In addition, home visits provide valuable insight into the patient's living conditions, dietary habits, and hygiene practices, allowing for personalized care planning (23). Collaboration with family members, caregivers, and primary healthcare providers ensures that oral care aligns with the patient's overall medical needs. Ultimately, home-based dental services promote comfort, dignity, and quality of life for dependent elderly individuals, bridging a critical gap in geriatric oral healthcare delivery. Such outreach-oriented care ensures inclusivity and continuity of treatment, particularly for underserved populations.

**Extending sponsored services:** Inclusion of old age homes in community-based oral health programs represents a step forward, ensuring access to dental care for elderly individuals. Many residents in such facilities face financial constraints, limited mobility, or lack of family support, which often leads to neglected oral health needs. By extending sponsored or free dental services to these institutions, healthcare providers can help bridge this gap and promote oral health as an essential component of overall well-being.

Under these initiatives, dental professionals can organize regular screening camps, preventive care sessions, and treatment programs within old-age homes. Services may include oral examinations, denture care, scaling, fluoride applications, and minor restorative procedures. Health education sessions for caregivers and residents can further encourage daily oral hygiene maintenance and early reporting of dental problems (21).

Collaborations with governmental bodies, NGOs, dental institutions, and private sponsors can sustain these programs through funding, materials, and professional support. Such outreach not only enhances the oral and systemic health of elderly residents but also reinforces the social responsibility of the dental profession. Ultimately, integrating old age homes into sponsored dental care programs fosters inclusivity, dignity, and improved quality of life for one of the most vulnerable segments of society (11).

## Policy and institutional implications

### Policy translation

Including old age homes within community-based oral health programs necessitates translation of geriatric oral health needs into actionable public health policies. National and state oral health policies should explicitly recognize institutionalized elderly populations as a priority group, similar to maternal and child health cohorts. Integrating oral health services for old age homes into existing frameworks such as the National Oral Health Programme (NOHP), National Programme for Health Care of the Elderly (NPHCE), and primary healthcare outreach can facilitate systematic service delivery. Policy mandates for periodic dental screening, preventive care, and referral pathways for elderly residents can ensure continuity of care rather than sporadic interventions (25).

### Curricular integration

Institutionalizing geriatric oral health care within dental education curricula is essential to building a competent workforce. Undergraduate and postgraduate dental programs should integrate structured training modules on geriatric dentistry, including clinical postings in old age homes and community outreach settings. Such exposure enhances students' understanding of age-related oral diseases, systemic comorbidities, and the social determinants affecting elderly health (26). Service-learning models, in which outreach activities are linked to academic credit, can strengthen student participation while reinforcing the ethical and social responsibilities of the dental profession. Sustainable financing is critical for the success of sponsored dental services in old-age homes. Funding can be mobilized through a mix of governmental allocations, public–private partnerships (PPPs), corporate social responsibility (CSR) initiatives, and NGO-supported grants. Inclusion of geriatric oral health services under publicly funded insurance or welfare schemes would further reduce out-of-pocket expenditure for elderly residents. Dental colleges and institutions can also be incentivized through funded outreach programs, ensuring both service provision and academic engagement. Transparent budgeting and outcome-based funding models can enhance accountability and long-term viability.

### Long-term sustainability

For long-term sustainability, these initiatives must transition from isolated outreach camps to institutionalized programs with defined monitoring and evaluation frameworks. Establishing partnerships between old age homes and nearby dental institutions or primary health centres can ensure regular follow-up and continuity of care. Training caregivers in basic oral hygiene practices and early identification of oral problems further strengthens sustainability at the grassroots level. Data generated from these programs can inform policy refinement, resource allocation, and research on geriatric oral health outcomes. Ultimately, embedding oral health services for the elderly within broader public health and social welfare systems promotes inclusivity, dignity, and an improved quality of life for aging populations.

### Limitations and possible challenges in implementation

Despite the clear benefits of extending sponsored dental services to old age homes, several limitations and implementation challenges must be acknowledged. Logistical constraints such as inadequate infrastructure within old age homes, limited availability of dental equipment, and the need for portable dental units may restrict the scope of on-site services. Workforce shortages, particularly a lack of trained professionals in geriatric dentistry, can further limit service delivery. Additionally, variability in residents' systemic health status, cognitive impairment, and polypharmacy complicates clinical decision-making and may restrict the feasibility of certain dental procedures.

Financial sustainability remains a key challenge, as reliance on short-term grants, CSR funding, or voluntary services may lead to discontinuity of care. Coordination between healthcare providers, old age home administrators, and policymakers can also be fragmented, resulting in inconsistent follow-up and referral pathways. Furthermore, cultural beliefs, low perceived priority of oral health among elderly individuals, and caregiver time constraints may reduce acceptance and adherence to recommended oral hygiene practices. These limitations highlight the need for structured planning, intersectoral collaboration, and integration of oral health into routine geriatric care services (27).

### Ethical and social aspects

Ethical considerations are central to the implementation of community-based oral health programs in senior care facilities. Informed consent must be obtained from all participating residents, with special provisions for individuals with cognitive impairment or reduced decision-making capacity, for whom consent should be sought from legally authorized representatives in accordance with relevant ethical guidelines. Clear communication regarding the nature of services, potential risks, benefits, and alternatives should be ensured in a language and format understandable to elderly participants (28).

Confidentiality and privacy must be safeguarded during clinical examinations and data collection, with secure handling of health records and anonymization of data used for research or program evaluation. Equity in service provision is essential, ensuring that all residents, irrespective of socioeconomic status, gender, or physical dependency, have equal access to care.

From a social perspective, these programs contribute to reducing health disparities and social neglect among institutionalized elderly populations, reinforcing dignity, autonomy, and social inclusion. Acknowledgment of institutional partnerships, governmental agencies, NGOs, funding bodies, and dental institutions involved in program implementation is ethically important to maintain transparency and accountability. Such recognition also encourages sustained collaboration and support, strengthening the credibility and long-term impact of geriatric oral health initiatives.

## Conclusion

The intersection of aging and oral health is a critical yet under-addressed area in global healthcare. The proposed interprofessional model fosters a paradigm shift—from treating disease in isolation to promoting holistic, preventive, and compassionate care. By integrating expertise from multiple disciplines, the model reimagines geriatric dentistry as an integral part of healthy aging. Policymakers, educators, and healthcare leaders are urged to embrace and scale such integrated frameworks to ensure that oral health becomes a central pillar of comprehensive geriatric well-being.

## Data Availability

The original contributions presented in the study are included in the article/Supplementary Material, further inquiries can be directed to the corresponding author.
